# Multi-modal diffuse optical spectroscopy for high-speed monitoring and wide-area mapping of tissue optical properties and hemodynamics

**DOI:** 10.1117/1.JBO.26.8.085002

**Published:** 2021-08-13

**Authors:** Jesse H. Lam, Brian Hill, Timothy Quang, Robert Amelard, Sehwan Kim, Hossein S. Yazdi, Robert V. Warren, Kyle B. Cutler, Bruce J. Tromberg

**Affiliations:** a University of California, Irvine, Beckman Laser Institute, Department of Biomedical Engineering, Irvine, California, United States; b Dankook University, Beckman Laser Institute Korea, School of Medicine, Cheonan, Chungnam, Republic of Korea; c National Institutes of Health, National Institute of Child Health and Human Development, Bethesda, Maryland, United States; d KITE-Toronto Rehabilitation Institute, University Health Network, Toronto, Ontario, Canada; e Schlegel-UW Research Institute for Aging, Waterloo, Ontario, Canada; f National Institutes of Health, National Institute of Biomedical Imaging and Bioengineering, Bethesda, Maryland, United States

**Keywords:** diffuse optical spectroscopy, imaging, chromophore, near-infrared, tissue metabolism

## Abstract

**Significance:** Diffuse optical spectroscopic imaging (DOSI) is a versatile technology sensitive to changes in tissue composition and hemodynamics and has been used for a wide variety of clinical applications. Specific applications have prompted the development of versions of the DOSI technology to fit specific clinical needs. This work describes the development and characterization of a multi-modal DOSI (MM-DOSI) system that can acquire metabolic, compositional, and pulsatile information at multiple penetration depths in a single hardware platform. Additionally, a 3D tracking system is integrated with MM-DOSI, which enables registration of the acquired data to the physical imaging area.

**Aim:** We demonstrate imaging, layered compositional analysis, and metabolism tracking capabilities using a single MM-DOSI system on optical phantoms as well as *in vivo* human tissue.

**Approach:** We characterize system performance with a silicone phantom containing an embedded object. To demonstrate multi-layer sensitivity, we imaged human calf tissue with a 4.8-mm skin-adipose thickness. Human thenar tissue was also measured using a combined broadband DOSI and continuous-wave near-infrared spectroscopy method (∼15  Hz acquisition rate).

**Results:** High-resolution optical property maps of absorption ($\mu_{a}$) and reduced scattering (${\mu_{s}}^{\prime }$) were recovered on the phantom by capturing over 1000 measurement points in under 5 minutes. On human calf tissue, we show two probing depth layers have significantly different (p<0.001) total-hemo/myoglobin and ${\mu_{s}}^{\prime }$ composition. On thenar tissue, we calculate tissue arterial oxygen saturation, venous oxygen saturation, and tissue metabolic rate of oxygen consumption during baseline and after release of an arterial occlusion.

**Conclusions:** The MM-DOSI can switch between collection of broadband spectra, high-resolution images, or multi-depth hemodynamics without any hardware reconfiguration. We conclude that MM-DOSI enables acquisition of high resolution, multi-modal data consolidated in a single platform, which can provide a more comprehensive understanding of tissue hemodynamics and composition for a wide range of clinical applications.

## 1 Introduction

Diffuse optical spectroscopic imaging (DOSI) is a versatile, non-invasive technology that is sensitive to tissue composition and hemodynamics. There is extensive literature describing the wide variety of clinical applications for DOSI ranging from breast cancer to exercise physiology.^1^^–^^7^ For breast cancer applications, sparsely sampling points on a discrete grid with a handheld probe enables a clinician to monitor breast tumor hemodynamics in response to neoadjuvant chemotherapy.^1^^,^^4^^,^^8^^,^^9^ Characterization of different tissue layers with multiple source-detector separations, such as breast adipose and the underlying tumor, can provide additional, unique insights.^10^^–^^14^ In previous work, using a DOSI-guided differential diagnosis method, the broadband spectral differences were compared between a breast with a benign or malignant tumor and the contralateral normal breast to demonstrate that analysis of multiple tissue layers with DOSI may supplement standard-of-care methods to determine tumor malignancy.^5^ DOSI can also be applied as a fixed-position, time series measurement (e.g., to monitor tissue hemodynamics). Optically obtained hemodynamic biomarkers have been identified as key indicators of hemorrhage and resuscitation.^15^^–^^19^ While DOSI has been a valuable complement for a wide variety of clinical applications, advancement of the instrumentation to collect multiple modalities and accurately track handpiece position in a single device is important for constrained clinical settings, capturing faster physiological signals, and for rapid visualization of the images corresponding to the physical, mapped area.

There is a wealth of metabolic, pulsatile, and compositional information that can be quantified with DOSI. For example, digital DOSI (dDOSI), has been recently developed which can collect data at far greater speeds than previously reported.^20^^,^^21^ A key advantage of dDOSI is its high acquisition speed which enables higher fidelity wide-area imaging capabilities and resolution of pulsatile hemodynamics. Resolving pulsatile hemodynamics enables calculation arterial oxygenation (SaO2),venous oxygenation (SvO2), and oxygen extraction fraction (OEF) which are fundamental physiological parameters related to oxygen supply and delivery.^22^^–^^25^ The OEF is also needed to calculate the tissue metabolic rate of oxygen consumption (tMRO2), which is a more direct measure of tissue metabolism compared to hemoglobin or oxygenation-based secondary biomarkers.^24^^,^^26^ Current approaches have been configured to interrogate a single tissue depth, but interrogation of metabolic, pulsatile, and compositional information at multiple tissue depths would be invaluable for characterizing heterogeneous tissues.

Considering these recent technological advancements, we developed a multi-modal DOSI system (MM-DOSI) that can acquire metabolic, compositional, and pulsatile information at multiple penetration depths in a single hardware platform. Additionally, we integrate a 3D tracking system to map the acquired images back onto the physical imaging area, enabling active feedback at the point-of-care. Improving the capabilities of DOSI can reduce the amount of time needed for data collection and provide greater information content by use of MM data. These modes can be selectively switched allowing for data collection at varying spatial and temporal scales on patients in one session. We validate this system in a series of optical phantom experiments followed by *in vivo* human measurements to demonstrate the performance of each individual modality.

## 2 Materials and Methods

### 2.1 DOSI Instrumentation

DOSI is a well-established quantitative optical technique which utilizes frequency-domain photon migration (FDPM) and continuous wave (CW) methods to recover absorption ($\mu_{a}$) and reduced scattering coefficients (${\mu_{s}}^{\prime }$) across the near-infrared spectrum (600 to 1000 nm). The theory and implementation of DOSI has been extensively reported in previous literature.^27^^–^^30^ Briefly, FDPM analyzes signals from discrete near-infrared light sources such as laser diodes intensity modulated in the MHz to GHz regime. By comparing the amplitude decay and phase shift to a reference as a function of frequency, the $P_{1}$ semi-infinite approximation for the radiative transport equation can be fit to the data and recover optical properties.^28^^,^^31^^–^^34^ Given a sufficient number of wavelengths, concentrations of tissue chromophores oxyhemoglobin (HBO2), deoxyhemoglobin (HbR), tissue water ($H_{2}O$), and tissue lipid (FAT) present in turbid media can be quantified by least-squares fitting using known extinction coefficients.^27^ It should be noted that in this work, effects of myoglobin absorption were not separated from hemoglobin. Thus, data shown for highly muscular tissues, such as thenar, should be considered a mixture of hemoglobin and myoglobin.^7^^,^^35^^,^^36^ For these tissues, we follow the convention of oxygenated hemo+myoglobin (HbMbO2), deoxygenated hemo + myoglobin (HbMbR), and total hemo+myoglobin (THbMb).^7^

The DOSI instrument houses two sets of three lasers with wavelengths centered at 727, 808, and 839 nm for a total of six lasers. Laser light is guided to tissue through custom 8-to-1 optical fiber bundles comprised of 400-μm optical fibers (R Specialty Optical Fibers LLC, Williamsburg, Virginia). A commercial laser diode controller (LDC-3916, Newport Corporation, Irvine, California) regulates laser operation while a network analyzer (TR1300/1, Copper Mountain Technologies, Indianapolis, Indiana) provides 3-dBm radio frequency (RF) modulation sequentially to each laser diode. Modulated laser light is collected through a 1-mm solid-core fiber optic cable coupled to an avalanche photodiode (APD) (S11519-30 APD with custom module, Hamamatsu Photonics K. K., Hamamatsu City, Japan). When broadband spectroscopy is desired, output from a tungsten-halogen lamp (HL-2000-FHSA, Ocean Optics Inc., Largo, Florida) and resulting diffuse reflectance is collected through a 1-mm solid-core fiber optic cable to a commercial spectrometer (HS2048XL-U2, Avantes, Apeldoorn, Netherlands). A consumer laptop computer (Razer Blade Pro 17, Razer Inc., San Francisco, California) operates the instrument and acquires data. Data analysis was performed using MATLAB (MathWorks, Natick, Massachusetts).

### 2.2 DOSI Acquisition Parameters

The network analyzer sequentially provides RF modulation to each laser diode in the system at a speed of approximately 150 to 200  μs/point. Taking careful consideration to avoid aliasing of the FDPM phase component, we empirically determined (data not shown) that 200 uniformly distributed sampling points spanning 50 to 500 MHz optimized network analyzer acquisition speed without sacrificing data quality. Other significant instrument delays in the system were due to the delayed-start logic of the laser diode controller. To bypass these delays each time a laser diode enabled, the average optical power level of each laser is maintained at a constant 30 mW throughout measurements, thus the acquisition speed of the system is ultimately limited by the maximum frequency sweeping time of the network analyzer. With optimized network analyzer measurement parameters, measurement rates of approximately 4 Hz are possible.

To capture pulsatile hemodynamics for the calculation of SaO2, SvO2, and tMRO2 at a single spatial location, we adapted a combined FDPM and CW near-infrared spectroscopy (CW-NIRS) method in which FDPM and reflectance data are asynchronously acquired. FDPM data is collected by the APD at a rate of ∼4  Hz while CW-NIRS data are collected by the spectrometer at a rate of ∼15  Hz. The combination of these two techniques allows us to quantify tissue optical properties and absolute hemoglobin concentration using FDPM while more rapid pulsatile fluctuations between FDPM datapoints are filled in by CW-NIRS. Because the modulation rate of the laser light is in the MHz regime, the intensity modulation is not perceivable by a spectrometer operating at tens of Hz. Thus, modulated laser light detected by the spectrometer can be effectively considered as CW. Tissue chromophores are extracted from the DOSI measurement using linear, least-squares fitting^27^ and the differential path length factor method when handling the CW-NIRS data.^37^^–^^40^

Table 1 summarizes the various measurement configurations of the optimized DOSI system. In broadband mode, the instrument can acquire data at ∼0.5  Hz with three laser diodes and a tungsten-halogen broadband lamp. The broadband configuration is unchanged from previous reported iterations of the DOSI device, with the capture rate dependent on the integration time of the spectrometer. With the added improvements, the instrument can acquire data at a rate of 4 Hz with three laser diodes in scanning mode, 2 Hz when expanded to all six laser diodes in dual-channel mode or 15 Hz when utilizing three diodes in the asynchronous FDPM and CW-NIRS pulsatile mode. Depending on the clinical application, these different measurement modes can be selected in software while maintaining the same hardware configuration. More technical details about the instrument operation can be found in Fig. S1 in the Supplementary Material.

**Table 1:** DOSI modalities and type of information collected by each mode. The modes can be selected on the fly to facilitate multi-modality data collection in a single measurement session. Capture rate refers to the speed at which each data point is acquired.

MM-DOSI measurement modes				
	Broadband	Scanning	Dual channel	Pulsatile
Capture rate	0.5 Hz	4 Hz	2 Hz	15 Hz
Quantitative HBO2, HBR	✓	✓	✓	✓
Pulsatile Hemodynamics	—	—	—	✓
Quantitative H2O, FAT	✓	—	—	—
Wide-area imaging	✓	✓	—	—
Multiple depth sampling	—	✓	✓	
${\mu_{s}}^{\prime }(\lambda )$	✓	✓	✓	✓

### 2.3 Optical Phantom Fabrication

A silicone-based solid optical phantom with a suspended equilateral cross-shaped silicone inclusion was fabricated for scanning mode imaging experiments. Dimensions of the inclusion were 5.1 cm across and 1.9-cm thick. Our tissue-simulating silicone phantom construction process has been described previously.^41^ Briefly, we used Nigrosin (Sigma-Aldrich, St. Louis, Missouri) and 44-μm particle diameter ${\mathrm{TiO}}_{2}$ powder (Loudwolf, Dublin, California) for the absorbing and scattering agents, respectively. A separate reference phantom was made from the same batch used to make the inclusion. The silicone was cured for a minimum of 24 h. To fabricate the final phantom with the suspended inclusion, the cured cross-shaped inclusion was submerged and suspended using pins at the desired depth within the background solution while it solidified. After 24 h, the pins were removed. The inclusion was suspended at a surface-to-surface depth of 6 mm. Optical properties of the pure phantom background ($\mu_{a}=0.007\;{\mathrm{mm}}^{-1}$, ${\mu_{s}}^{\prime }=0.62\;{\mathrm{mm}}^{-1}$) and pure inclusion ($\mu_{a}=0.033\;{\mathrm{mm}}^{-1}$, ${\mu_{s}}^{\prime }=0.32\;{\mathrm{mm}}^{-1}$) were characterized using DOSI at 808 nm, giving an approximate inclusion-to-background contrast ratio of 4.7 and 0.5 for absorption and scattering, respectively.

### 2.4 3D Tracking System Integration

**Fig. 1:**
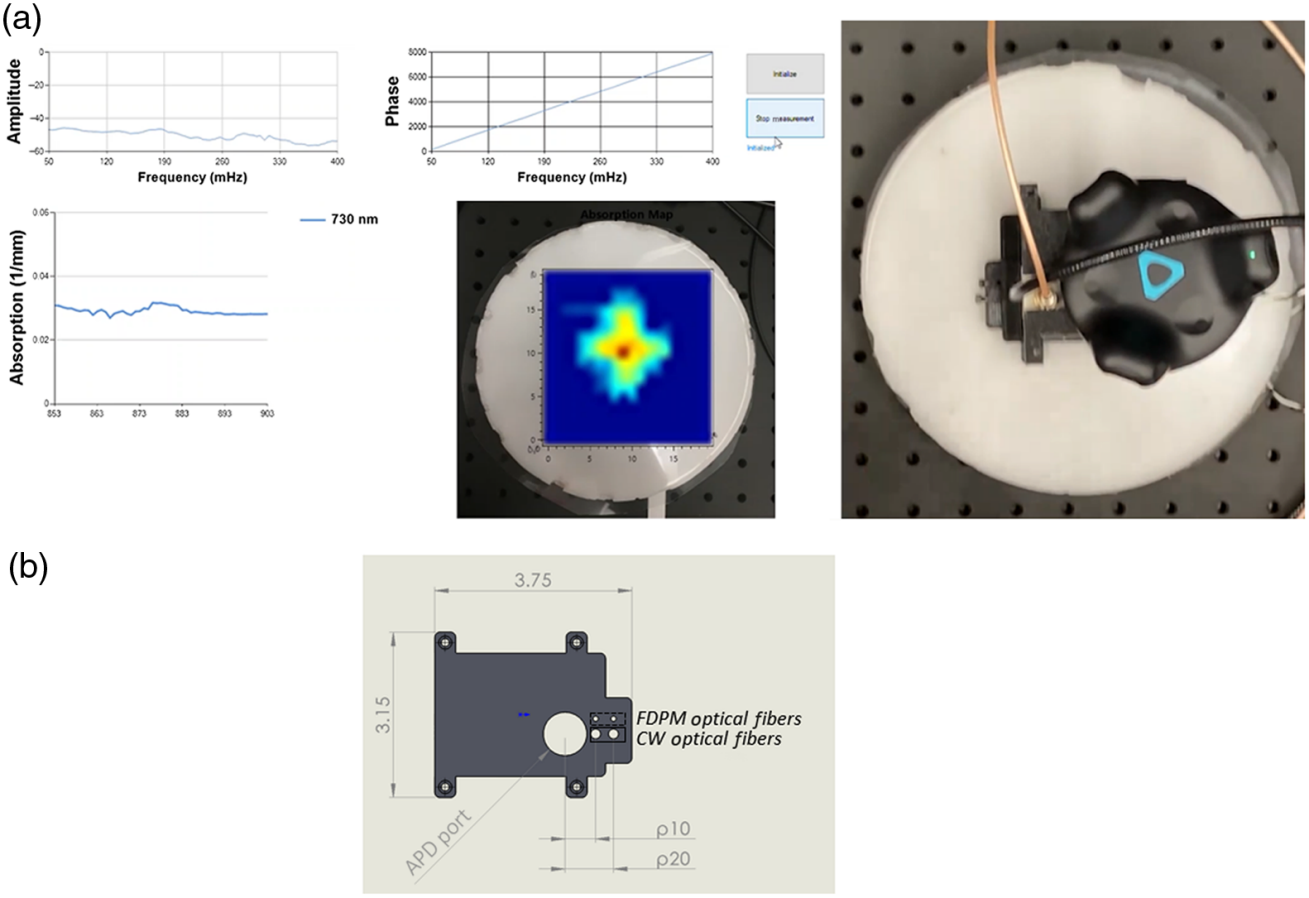
Scanning measurements are achieved using a probe with integrated motion tracker. (a) Screen capture of the real-time imaging (Video 1) of a 12  cm×12  cm area of phantom with embedded inclusion (left) while DOSI handpiece is scanned across phantom surface (right). The probe with attached source optical fiber, detection optical fiber, and HTC VIVE Tracker is shown on the right. The HTC VIVE Lighthouse was placed approximately 1 meter above the scanning surface (Lighthouse not captured in image frame). (b) Bottom view of a probe with an APD port and two options to adjust position of the FDPM and CW source optical fibers. CW light is collected using an optical fiber passed through the APD case. Dimensions are in inches. The VIVE tracker is mounted on top of the APD module (Video 1, MP4, 10.5 MB [URL: https://doi.org/10.1117/1.JBO.26.8.085002.1]).

To accurately orient the acquired DOSI data with respect to the physical imaging area, an HTC VIVE Tracker (HTC Corporation, Taoyuan City, Taiwan) recorded the motion of the probe during image acquisition. The tracking system consisted of a “Lighthouse” base station with infrared laser emitter and a detection unit. The VIVE Tracker utilizes a time-of-flight approach and distributed photodiodes around the detection unit to calculate position and orientation.^42^ The base station was positioned ∼1  m above the measurement plane while the detection unit was affixed to the probe. Figure 1 (Video 1, MP4, 10.5 MB) shows a real-time demonstration of MM-DOSI collecting data with the HTC VIVE tracker while scanning over the inclusion phantom described in Sec. 2.3. Real-time estimation allows users to identify and focus data collection on regions of enhanced contrast. Higher-resolution images of the same scan can then be processed offline. Data was captured at ∼4  Hz with the probe manually raster scanned throughout the acquisition area. The phantom was measured with the probe SDS configured at 8 mm ($\rho_{8}$). To compare the difference in image resolution to previously described discrete grid DOSI mapping applications, the data were sparsely sampled to simulate a 1-cm spacing grid. For visualization, data were interpolated using a cubic spline method.

### 2.5 In Vivo Imaging

To demonstrate scanning mode imaging capabilities, a 5  cm×8  cm area on the medial gastrocnemius (i.e., calf) of a healthy human male subject was imaged at an SDS of 10 mm ($\rho_{10}$) and 20 mm ($\rho_{20}$). The subject was instructed to sit comfortably in a chair and rest his extended leg on a table while being imaged with the probe held similarly as shown in Fig. 1. We measured skin and adipose tissue thickness with ultrasound (SonoSite Titan, Fujifilm SonoSite, Bothell, Washington). Informed consent was provided to the subject, and procedures were carried out under UC Irvine IRB-approved protocol (2004-3626).

### 2.6 In Vivo Dual Layer Measurements

Differences in the hemodynamic response at different tissue depths (adipose versus mixed adipose-muscle) were demonstrated by an arterial occlusion measurement with a dual-channel probe affixed to the center of the same calf area. Data were captured in dual-channel mode. The probe was configured to measure at $\rho_{10}$ and $\rho_{20}$. After 90 s of baseline, a rapid occlusion cuff (AG101 pump with E20 regulator, Hokanson Vascular, Bellevue, Washington) was inflated to 200 mmHg for 90 s, and then released for 90 s. Tissue composition (e.g., FAT, H2O, and ${\mu_{s}}^{\prime }$) was also obtained with broadband mode to more accurately quantify absolute hemoglobin values.^30^ These measurements were obtained in a 3  cm×2  cm grid with 1-cm spacing at the same SDS configurations. The hemodynamics were smoothed with a second-order median filter as the acquisition speed in this mode was not fast enough to capture pulsatile signals, and evaluation of the overall dynamics was ultimately desired.

### 2.7 In Vivo Rapid Hemodynamics

Pulsatile hemodynamics were captured at 15 Hz to assess tissue SaO2, SvO2, and tMRO2 on a one-layer, homogenous model. This was achieved by a point measurement on the thenar muscle during an arterial occlusion of the same subject. Broadband mode provided absolute quantification of tissue chromophores while high-speed data were captured by pulsatile mode. The differential path length factor for the recovery of hemodynamics was calculated (mean and standard deviation of 2.71±0.41) with the absolute optical properties.^37^^,^^38^ The measurement protocol consisted of a 60 s baseline, 90 s occlusion at 200 mmHg, and a 90 s recovery. tMRO2 was calculated for the entire measurement timeline using only DOSI parameters and compared against tMRO2 utilizing relative blood flow (rBF) measured by diffuse correlation spectroscopy (DCS). tMRO2 calculations were derived from methods described previously.^43^ An additional thenar occlusion was performed, and BF was measured with DCS at $\rho_{24}$.

The DCS instrument introduces light into tissue from a 785 nm, long coherence length laser (Crystalaser, Nevada) with the optical output set to 30 mW. Four single photon counting modules (SPCM-AQRH-15-FC, Excelitas, Canada) connected to a 4-to-1 single-mode fiber bundle detect the diffuse light that exits the tissue. The digital outputs of the detectors are connected to an instrument timer/counterboard (PCIe6612, National Instruments, Austin, Texas) and captured by a custom LabVIEW interface (National Instruments, Austin, Texas) with an acquisition rate of 20 Hz. *tMRO2* was calculated with the following expression:

$$tMRO2=[O_{2}{]}_{art}\cdot \alpha \cdot rBF\cdot OEF,$$ (1)

where ${\left[O_{2}\right]}_{\text{art}}$ is the molar oxygen concentration in arterial blood, α is a calibration factor relating relative to absolute BF,^43^ *rBF* is the relative blood flow, and OEF is defined as

$$\mathrm{OEF}=\frac{{\left[O_{2}\right]}_{\text{art}}-{\left[O_{2}\right]}_{\text{vein}}}{{\left[O_{2}\right]}_{\text{art}}},$$ (2)

$${\left[O_{2}\right]}_{\text{art}}=4\cdot {\left[\mathrm{tHb}\right]}_{\text{art}}\cdot \mathrm{SaO}2,\;{\left[O_{2}\right]}_{\text{vein}}=4\cdot {\left[\mathrm{tHb}\right]}_{\text{vein}}\cdot \mathrm{SvO}2.$$

${\left[O_{2}\right]}_{\text{vein}}$ is the molar oxygen concentration in venous blood, while ${\left[\mathrm{tHb}\right]}_{\text{art}}$ and ${\left[\mathrm{tHb}\right]}_{\text{vein}}$ are the total intravascular hemoglobin concentration in the arterial and venous system, respectively. SaO2 and SvO2 are the arterial and venous oxygen saturation fractions, respectively. Intravascular hemoglobin in the circulatory system is assumed to be conserved during oxygen exchange, such that ${\left[\mathrm{tHb}\right]}_{\text{art}}={\left[\mathrm{tHb}\right]}_{\text{vein}}$. The expression for tMRO2 simplifies to

$$\mathrm{tMRO}2=4\cdot (\mathrm{SaO}2-S2)\cdot \alpha \cdot \mathrm{rBF}\cdot {\left[\mathrm{tHb}\right]}_{\mathrm{art}}.$$ (3)

The parameter α can be determined by tracking the rate of HbR accumulation during a zero-flow condition such as an arterial occlusion. Using the relationship between tMRO2 and HbR (i.e. ${\mathrm{tMRO}}_{2}=4\cdot \frac{d[\mathrm{HbR}]}{\mathrm{dt}}$) for the first minute of an arterial occlusion,^43^ α can be calculated and substituted into Eq. (3) to obtain

$$\mathrm{tMRO}2=4\cdot (\mathrm{SaO}2-\mathrm{SvO}2)\cdot \frac{\frac{d[\mathrm{HbR}]}{\mathrm{dt}}}{{\left(\mathrm{SaO}2-\mathrm{SvO}2\right)}_{\mathrm{bl}}\cdot {\mathrm{rBF}}_{\mathrm{bl}}}\cdot \mathrm{rBF},$$ (4)

where ${\left(\mathrm{SaO}2-\mathrm{SvO}2\right)}_{\mathrm{bl}}$ is the average difference between SaO2 and SvO2 during baseline, and ${\mathrm{rBF}}_{\mathrm{bl}}$ is the average rBF during baseline.

SaO2 can be optically measured using pulsatile mode by isolating the pulsatile hemoglobin signal and calculating arterial oxygenation, represented by the equation, $\mathrm{SaO}2=\frac{\mathrm{HbO}2_{\text{pulse}}}{{\mathrm{tHb}}_{\text{pulse}}}$ with $tHb_{\text{pulse}}$ as the arterial total hemoglobin calculated by the sum of pulsatile oxyhemoglobin ($HbO2_{\text{pulse}}$) and pulsatile deoxyhemoglobin (${\mathrm{HbR}}_{\text{pulse}}$).^44^ SvO2 can be calculated by assuming an arterial blood volume fraction (β) of 0.3 based on previous literature.^14^^,^^45^ The fraction of arterial total hemoglobin (${\mathrm{tHb}}_{\text{art}}$) and oxyhemoglobin (${\mathrm{HBO}2}_{art}$) including pulsatile and non-pulsatile arterial blood volumes was estimated with the equations ${\mathrm{tHb}}_{\mathrm{art}}=\beta \cdot \mathrm{tHb}$ and $\mathrm{HbO}2_{\text{art}}={\mathrm{tHb}}_{\text{art}}\cdot \mathrm{SaO}2$, respectively. Finally, ${\mathrm{SvO}}_{2}$ can be calculated by removing the contribution of arterial hemoglobin from the overall tissue saturation:

$$\mathrm{SvO}2=\frac{\mathrm{HbO}2-\mathrm{HbO}2_{\text{art}}}{\mathrm{tHb}-{\mathrm{tHb}}_{\text{art}}}.$$ (5)

Here, rBF is a non-quantitative measure of rBF and was estimated using pulsatile mode parameters. The amplitude of measured pulsatile hemoglobin was defined as proportional to the rBF:

$$rBF\propto tHb_{\text{Pulse}}=HbO2_{\text{Pulse}}+HbR_{\text{Pulse}}.$$ (6)

## 3 Results

### 3.1 Phantom Imaging

**Fig. 2:**
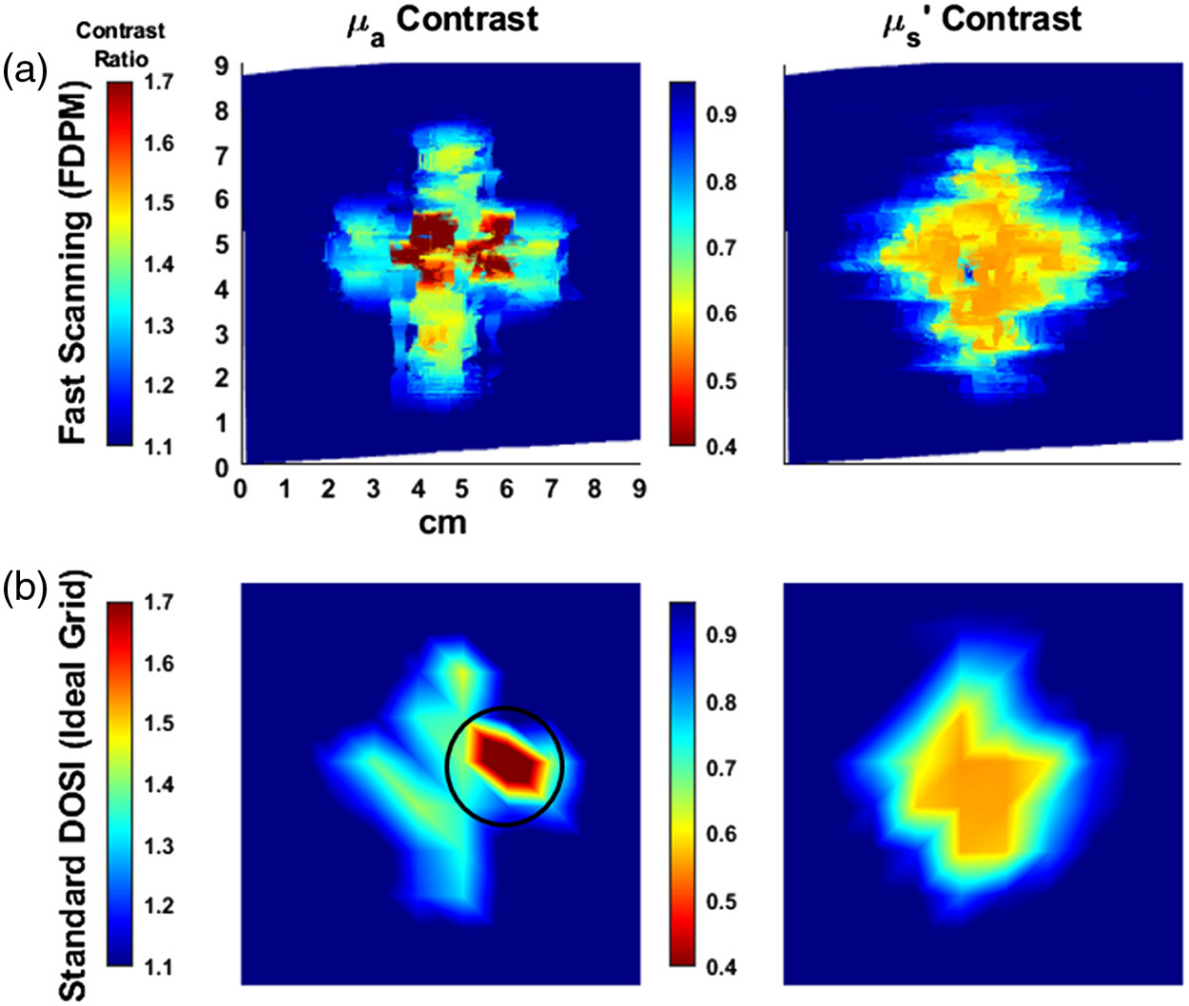
Pixel to background contrast ratio images of the absorption and reduced scattering maps at 808 nm at $\rho_{8}$ (in ${\mathrm{mm}}^{-1}/{\mathrm{mm}}^{-1}$ units). (a) Images were captured by manually raster scanning the surface of the phantom within a 12  cm×12  cm defined perimeter. The background was calculated by averaging the data using 1 cm squares at the four corners of the image. (b) Sparse-sampled data simulating a 1-cm grid image as captured in a discrete grid acquisition. Only one of two pinholes were detected (circled) due to the location of the sampling points.

Figure 2 shows images of the $\mu_{a}$ and ${\mu_{s}}^{\prime }$ signal-to-background contrast ratios for the 808-nm wavelength along the 6-mm deep inclusion phantom generated using scanning mode configured at $\rho_{8}$. To generate the contrast ratio maps, each pixel value was divided by the background, defined as the average value at 1  cm×1  cm regions from the four corners of the image. No additional filtering algorithm was applied to the image. A complete scan of the area yielded 1235 data points and was acquired within 4.8 min. Previously described, standard, discrete grid DOSI imaging approaches yield 100 to 200 data points over a span of 20 to 40 min. The pin holes through the surface of the phantom appear in the images as areas of increased absorption. Figure 2(b) shows sparse-sampled data using a 1-cm grid which simulates an image that would be acquired with discrete grid DOSI approach. Sparse-sampled points were selected using nearest integer values with no assumptions about the inclusion shape.

### 3.2 In Vivo Imaging and Hemodynamics

**Fig. 3:**
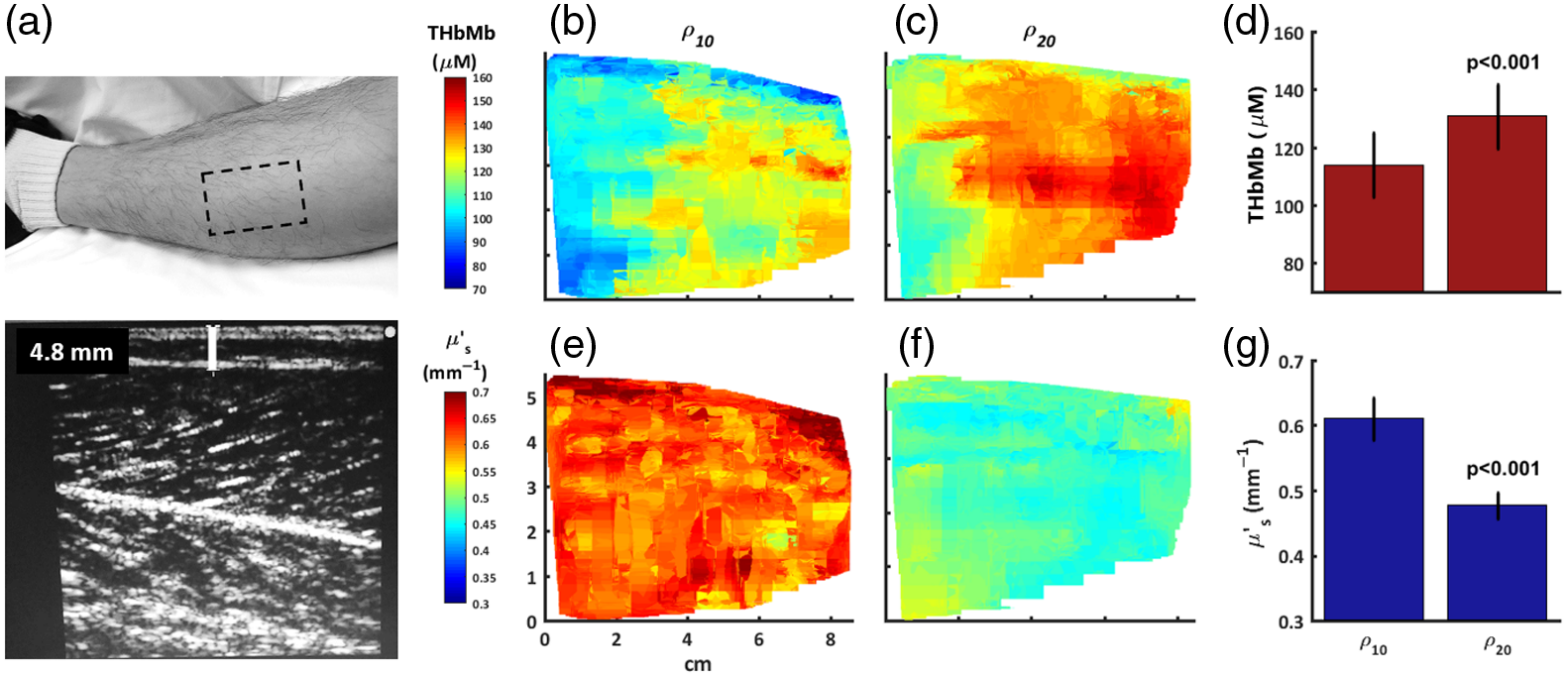
The calf of a healthy male subject imaged at $\rho_{10}$ and $\rho_{20}$. (a) Photograph of the 5  cm×7  cm imaging area (dotted box). Ultrasound at the center of the imaged area measured a skin-adipose layer of 4.8 mm. (b) THbMb of the imaged area at $\rho_{10}$ with an average value of 110.4±14.3  μM (c) THbMb of the imaged area at $\rho_{20}$ with an average value of 126.8±12.4  μM. (d) The mean and standard deviation of THbMb for $\rho_{10}$ and $\rho_{20}$. The rank sum test supports that the THbMb distributions were different (p<0.001). (e) $\mu_{s}n$ of the imaged area at $\rho_{10}$ with an average value of $0.63\pm 0.05\;{\mathrm{mm}}^{-1}$; (f) $\mu_{s}n$ of the imaged area at $\rho_{10}$ with an average value of $0.48\pm 0.03\;{\mathrm{mm}}^{-1}$. (g) The mean and standard deviation of ${\mu_{s}}^{\prime }$ for $\rho_{10}$ and $\rho_{20}$. The rank sum test supports that the ${\mu_{s}}^{\prime }$ distributions were different (p<0.001).

The calf of a healthy male subject was imaged twice using scanning mode with the probe SDS at $\rho_{10}$ and $\rho_{20}$. For each image set, ∼500 data points were collected over a 5  cm×8  cm area in a time span of 2 min. Figure 3(a) shows the perimeter of the imaging area, with a skin-adipose layer thickness of 4.8 mm as seen in the ultrasound image. Figures 3(b) and 3(c) shows chromophore maps of THbMb for $\rho_{10}$ (110.4±14.3  μM) and for $\rho_{20}$ (126.8±12.4  μM), respectively. Results of the Wilcoxon rank sum test suggests the two *THbMb* distributions were significantly (p<0.001) different [Fig. 3(d)]. Similarly, Figs. 3(e) and 3(f) show maps of ${\mu_{s}}^{\prime }$ for $\rho_{10}$ and $\rho_{20}$, respectively and the mean value of each map was $0.63\pm 0.05\;{\mathrm{mm}}^{-1}$ and $0.48\pm 0.03\;{\mathrm{mm}}^{-1}$, respectively. Again, the Wilcoxon rank sum test supported the hypothesis of a significant (p<0.001) difference between the two $\mu_{s}n$ distributions [Fig. 3(g)]. TMbHb values as depicted were calculated with consideration of the FAT and H2O tissue values provided by broadband. The average tissue H2O fraction for $\rho_{10}$ and $\rho_{20}$ was 46.6±3.5% and 48.8±3.7%, respectively, and the average tissue FAT fraction was 77.3±5.7% and 66.5±6.2%, respectively.

**Fig. 4:**
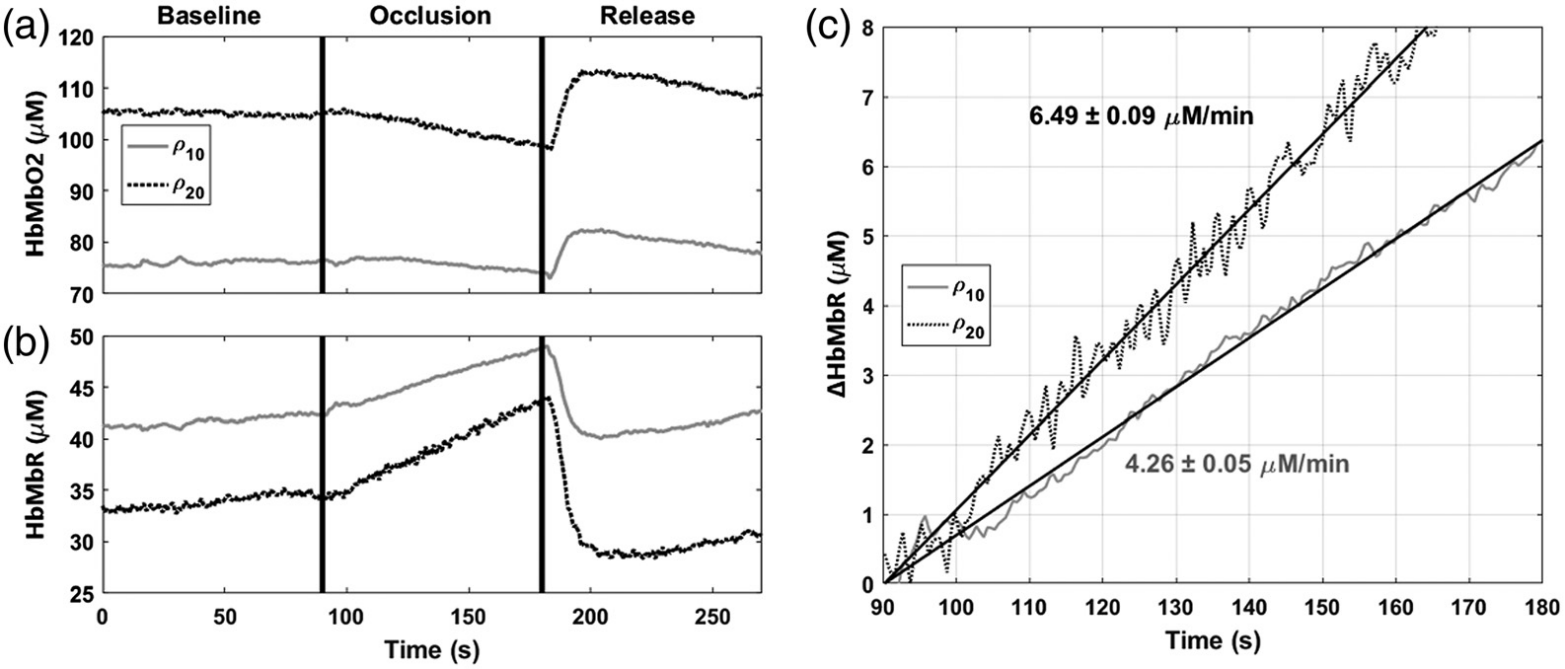
A calf occlusion was performed while measuring $\rho_{10}$ and $\rho_{20}$ in a single probe. (a) HbMbO2 dynamics at $\rho_{10}$ (black dashed) and $\rho_{20}$ (gray solid) during 90 s of occlusion and release. (b) HbMbR dynamics at $\rho_{10}$ and $\rho_{20}$. (c) The rate and 95% confidence interval of HbMbR conversion was calculated during the 90 s occlusion period.

An arterial occlusion was performed on the calf of the same subject and measured by dual-channel mode at $\rho_{10}$ and $\rho_{20}$. The measurement was taken at a single spatial location at the center of the imaging area from Fig. 3(a). The overall acquisition rate was 2 Hz. Figures 4(a) and 4(b) show the HbMbO2 and HbMbR dynamics for both SDS, respectively. We fit a line through the HbMbR trace during the 90 s occlusion period to calculate the rate of HbMbR accumulation. Figure 4(c) compares the linear fits between $\rho_{10}$ and $\rho_{20}$. During the occlusion phase, the slope of *HbMbR* at $\rho_{10}$ was 4.26  μM/min and 6.49  μM/min at $\rho_{20}$.

**Fig. 5:**
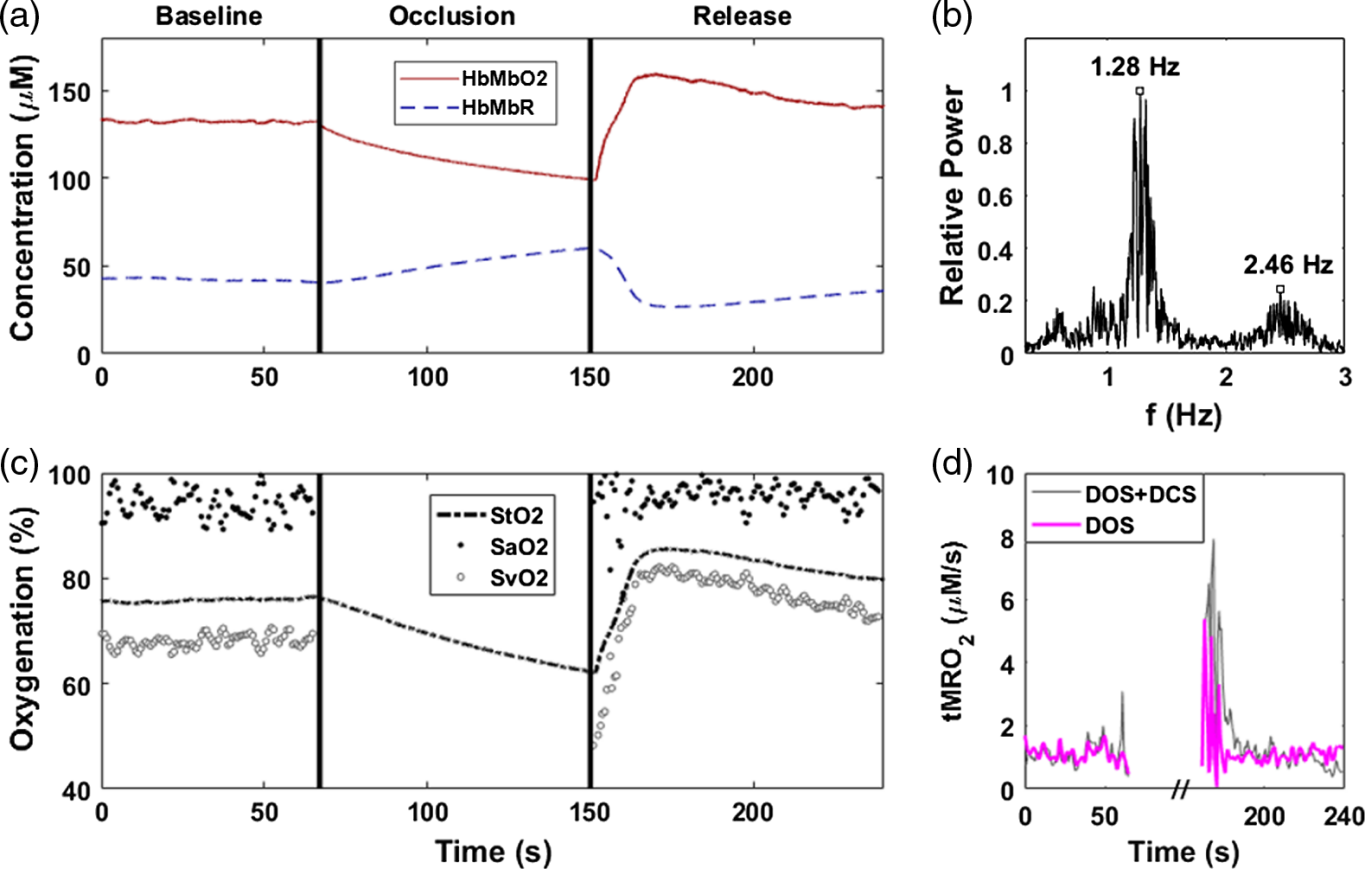
A thenar occlusion was performed on a healthy male subject. (a) HbMbO2 (red) and HbMbR (dashed, blue) dynamics are shown. The hemoglobin values were calculated provided H2O and FAT quantification by BB-DOSI. (b) Normalized frequency power spectrum with a 1.28-Hz peak representing heart rate and a 2.46-Hz peak for the first harmonic of the heart rate. (c) StO2 (dashed), SaO2 (black dots), and SvO2 (white circles) were calculated during baseline and release. (d) tMRO2 calculated using MM-DOSI (magenta line) and tMRO2 using BB-DOSI with DCS-provided flow from a secondary occlusion (gray line).

An occlusion on the thenar of the same subject was performed and measured using pulsatile mode at $\rho_{24}$. The thenar was selected due to the exceptionally thin skin-adipose thickness. The tissue area was assumed to be a single-layer, semi-infinite homogenous medium. broadband mode quantified the tissue composition, while pulsatile mode captured rapid hemodynamics assuming tissue FAT and H2O remained constant over the occlusion period. The overall data capture speed was 15 Hz. Hemoglobin quantification took into account a tissue *H2O* content of 83.8% and *FAT* content of 1.2%. Figure 5(a) shows the HbMbO2 and HbMbR dynamics for the occlusion measurement. Figure 5(b) shows the normalized frequency power spectrum of the pulsatile signal during baseline and after release in the heartbeat band. The heart rate of the subject was ∼1.28  Hz (77 beats per minute) with some small deviation throughout the four minute measurement. In addition, the second harmonic of the heartbeat can be recognized at 2.46 Hz. Figure 5(c) shows the tissue oxygen saturation ($\mathrm{StO}2=\frac{\mathrm{HbMbO}2}{\mathrm{THbMb}}\times 100$) and SaO2 as calculated over time. *rBF* was calculated using the pulsatile *tHb* signal captured using pulsatile mode. *rBF* can also be measured using DCS provided tissue optical properties from DOSI. Figure 5(d) compares the calculated tMRO2 using Eq. (4) with parameters solely recoverable by MM-DOSI against tMRO2 calculated with broadband mode augmented by DCS BF data. Overall dynamics were similar between the two tMRO2 measures: higher peak ${\mathrm{tMRO}}_{2}$ was reported by the DCS (7.9  μM/s) compared to MM-DOSI (5.4  μM/s).

## 4 Discussion

In this work, we introduce additional acquisition modalities to expand the information content of DOSI, enabling compositional, metabolic, and pulsatile information at higher speeds and multiple penetration depths. Additionally, we adapt and incorporate a commercially available tracking system to enable visualization of the acquired DOSI images correlating to the physical imaging area. The advantage of the single hardware configuration is that collection parameters can be selected at will to prioritize the desired information content for a given application. Different modalities will quantify different physiologic information, so the main technical challenge lies in weighing the trade-offs between acquisition speed and information content. For instance, while the dual-layer mode would supply information at multiple interrogation depths, the faster speed of the scanning mode would be better suited for applications requiring wide-area mapping. This expansion of different collection modes enables DOSI to meet the demands of a wide range of clinical applications. The integration of the tracking system is especially useful for rapid visualization of subsurface tumors and collecting data at multiple interrogation depths enable more accurate characterization of skeletal muscle hemodynamics. For this preliminary evaluation, we have demonstrated the MM-DOSI’s ability to scan and characterize wide areas of tissue, stratify responses from distinct tissue layers, and capture pulsatile hemodynamic information at a single spatial location.

### 4.1 Imaging Validation on Optical Phantoms

To demonstrate the imaging capabilities of the scanning mode, a phantom with a 6-mm deep, non-circular inclusion was scanned using a motion-tracked probe. The shape of the inclusion imaged at the 8-mm source-detector separation ($\rho_{8}$) was well-defined and in the expected shape for the $\mu_{a}$ contrast. Borders of the object were less defined for ${\mu_{s}}^{\prime }$, likely due to the lower contrast ratio between the background and inclusion as well as potential scattering interactions at the boundaries between the embedded object and background silicone. The surface holes on the phantom from the pins used to fix the inclusion into place were detected as regions of heightened attenuation for two reasons: (1) diffusive light leakage out of the phantom matrix into these air pockets, (2) coupling loss when the source or detection fiber was scanned directly over a pin hole. The pin holes were clearly visible in the image when scanning at $\rho_{8}$. Overestimation of absorption when either the source or detector optical fibers were positioned over the pinholes sometimes resulted in a distorted pinhole shape. The ability to distinguish the pinholes within the inclusion region suggests the capability to further delineate smaller heterogeneities within the heterogeneous phantom.

**Fig. 6:**
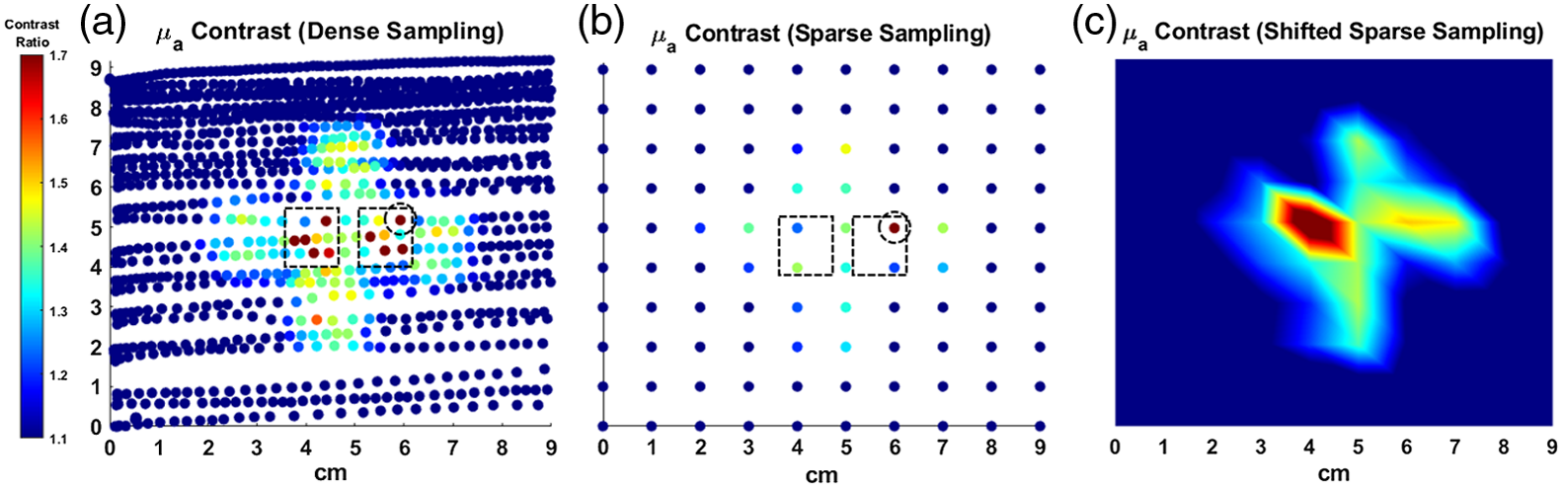
Pixel to background $\mu_{a}$ contrast ratio shown without spatial interpolation measured using 808 nm with the probe at $\rho_{8}$. (a) The dashed squares enclose high-contrast datapoints from the two pinholes seen in the high-density dataset. (b) For the left pinhole, high-contrast datapoints fell between the 1-cm spaced grid points, resulting in only the right pinhole appearing in the sparse sampled image. The dashed circle denotes a high-contrast datapoint from the right pinhole which was successfully captured by sparse sampling. However, only a single point at the far edge of the pinhole contrast region was captured. This resulted in an apparent translation of the pinhole position in the sparse sampled data, with the actual position of the pinhole more closely represented by the higher density data. (c) The sampling grid points can be shifted (−0.3  cm, −0.3  cm) to capture the left pinhole. However, doing so results in suboptimal sampling of the right pinhole as well as the inclusion edges. This further emphasizes that the placement of a sparse sampling grid can return highly variable images.

To accentuate improvements in image resolution using the MM-DOSI scanning mode, a sparse 1-cm spaced grid, akin to discrete-point DOSI imaging methods reported in previous works, was simulated by digitally selecting points from the denser dataset. In the sparse-sampled data, the four arms of the cross-shaped inclusion could be detected in the sparse data, although with some aberrations to the overall object shape. Two other major feature disparities were observed due to sampling differences between the dense and sparse datasets, as shown in Fig. 6. (1) The left pinhole was not visualized in the sparsely sampled data. This is because the high-contrast datapoints for the left pinhole fell between the sparse sampling points. (2) The right pinhole appeared to be translated in position when compared to the dense dataset. Due to the grid sparsity and placement, only the edge of the right pinhole was captured. This, in effect, moved the apparent location of the contrast region, when in actuality, most of the datapoints representing the right pinhole remained unsampled. Lastly, we show that shifting the sampling grid by (−0.3  cm, −0.3  cm) can allow us to capture the left pinhole. However, this results in suboptimal sampling of the right pinhole. We also observe that the shifted sampling grid is not well-aligned with the inclusion borders, leading to further distortion of the perceived inclusion shape. This further emphasizes that the placement of a sparse sampling grid can return highly variable images. While sparse sampling may be sufficient to detect some general features of a non-circular inclusion, we demonstrate that our MM-DOSI scanning method is particularly advantageous for enhancing the imaging resolution of absorbing inclusions as well as the detection of smaller heterogenous features within the inclusion structure.

### 4.2 Dual-Channel In Vivo Quantitative Composition Imaging and Hemodynamics

Demonstrating dual channel, two-layers of the calf of a healthy male subject were imaged at $\rho_{10}$ and $\rho_{20}$. Ultrasound and broadband mode provided the skin-adipose thickness and quantitative tissue composition, respectively. A survey of the ${\mu_{s}}^{\prime }$ and THbMb images concluded that $\rho_{10}$ and $\rho_{20}$ were probing significantly (p<0.001) different tissue compositions. As expected, the shallow layer contained more scattering components, likening to adipose tissue. Probing deeper, greater levels of *THbMb* were reported, likely due to the presence of more muscle mass. These findings were further corroborated when an arterial occlusion was performed on the same tissue site. During the arterial occlusion, there was a clear difference observed for the deoxygenation rate between the deep and shallow layers; the deeper tissue consumed oxygen 52% faster. This observation supports the finding that $\rho_{10}$ and $\rho_{20}$ were probing volumes comparable to adipose and adipose-muscle mixed tissue, respectively. Moving forward, due to the speed and ease of capturing multimodal datasets, it may be advantageous to simply image at multiple SDS on patients without significantly extending the measurement time. This may facilitate future opportunities where perturbations of layered tissues are studied, e.g., changes in top-layer adipose scattering^2^ as well as underlying muscle THbMb due to fitness training^7^ can both be tracked by this instrument.

Gentle probe-tissue compression combined with a relatively small scanning area resulted in an approximately flat scanning surface. Although the present work was proof-of-principle, future studies on more curved surfaces may incorporate profile-correction techniques. Approaches to generate 3D profiles of scanned tissues could potentially be adopted based on previously explored methods.^46^^,^^47^

### 4.3 Single-Layer In Vivo Quantitative Pulsatile Hemodynamics

To demonstrate pulsatile mode, thenar tissue was measured during an arterial occlusion at $\rho_{24}$ with H2O and FAT fractions of the tissue site quantified with broadband mode. Due to the exceptionally thin skin-adipose layer at the measurement site, the sampled tissue measured was approximated as a single homogenous muscle layer. We note that the presence of myoglobin may contribute to tissue oxygenation dynamics. Nevertheless, relative tMRO2 comparisons between MM-DOSI and DOSI-DCS still remain valid, as both techniques rely on a common occlusion factor, as described by Eq. (4).

SaO2 was separated from StO2 by assuming the pulsatile flow measured with pulsatile mode was dominated by arterial BF. By bandpass filtering the pulsatile data to the heart rate band, pulsatile hemoglobin within the arterial system was isolated. Using Eq. (6), we estimated rBF with ${\mathrm{tHb}}_{\text{Pulse}}$ which was calculated from pulsatile mode. ${\mathrm{HbO}2}_{\text{Pulse}}$ may also serve as a surrogate for *rBF* but could potentially be affected by the conversion of *HbO2* to *HbR* during metabolic activity. Thus, ${\mathrm{tHb}}_{\text{Pulse}}$ was selected as a surrogate for pulsatile BF. Applying Eq. (4), tMRO2 was then approximated at the same site using MM-DOSI data. To validate this approach, a second occlusion was performed using DCS at the same SDS. Optical properties from the broadband mode were used for the DCS BF calculations. tMRO2 calculated using DCS was comparable to the MM-DOSI approach. For the DOSI-DCS system, peak tMRO2 was 7.9  μM/s compared to 5.4  μM/s reported by MM-DOSI. This discrepancy can be explained by a variety of factors such as a slight probe placement offset, greater sensitivity of DCS to motion artifacts (e.g., during release of the occlusion cuff pressure) or the sequential sampling of the DOSI and DCS systems. For both instruments, the relaxation of tMRO2 to baseline in response to the brief 90 second occlusion was ∼20  s. These results suggest tMRO2 dynamics may be approximated using parameters solely provided by MM-DOSI. This approach relies on a DOSI device with sufficient acquisition speed as well as quantified tissue composition, further emphasizing the benefits of multiple modalities during a single measurement session.

## 5 Conclusion

To meet the demands of a range of clinical applications, we have presented the capabilities of MM-DOSI as a flexible clinical research tool. By optimizing the speed of our FDPM acquisition pipeline in conjunction with motion tracking technology, MM-DOSI allows for co-localized capture of tissue optical properties, hemodynamics, composition, and estimation of tMRO2 in a single measurement session using a single hardware configuration. The device was demonstrated on tissue-simulating optical phantoms with a buried inclusion as well as *in-vivo*. The higher acquisition speed improves resolution during imaging, enables monitoring of physiologic changes on a faster timescale and facilitates the recovery of pulsatile hemodynamics, rBF, and ultimately tMRO2 dynamics as a result of arterial occlusion. This was compared against tMRO2 using rBF provided by DCS during a second occlusion. On layered tissue, composition of mixed-shallow and mixed-deeper layers were recovered with dual-channel mode, suggesting constitutions similar to adipose and muscle, respectively. This provides more comprehensive characterization of tissue which may prove beneficial in studies monitoring disease or intervention progression. We show a single MM-DOSI device capable of data collection on multiple spatial and temporal fronts, potentially simplifying optical hardware requirements and maintaining the flexibility for a wide variety of clinical research applications.

## Code, Data, and Materials Availability

Data underlying the results presented in this paper are not publicly available at this time but may be obtained from the authors upon reasonable request.
