# Supplementary material for: Multi-modal diffuse optical spectroscopy for high-speed monitoring and wide-area mapping of tissue optical properties and hemodynamics
Source: J Biomed Opt. 2021 Aug 13;26(8):085002. doi: 10.1117/1.JBO.26.8.085002 (PMC8362892; doi:10.1117/1.JBO.26.8.085002)
Supplement: Supplementary file 1 [file JBO_026_085002_SD002.pdf]

Supplementary Material

Instrument diagram

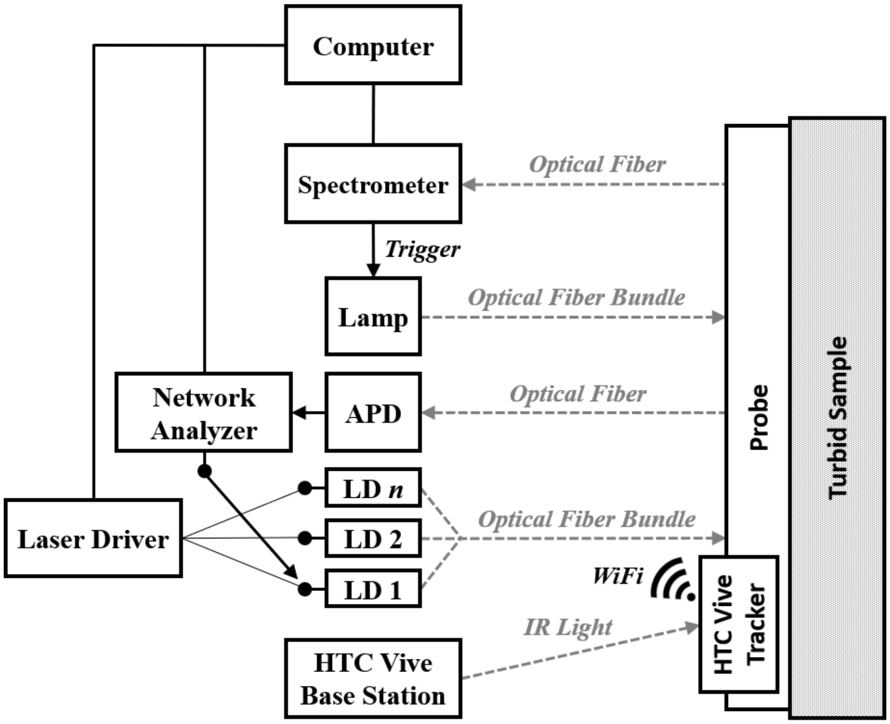

Fig. S1 System block diagram of MM-DOSI. Solid black lines signify electrical signals, while dashed gray lines signify optical signals. Core components of this instrument are similar to those reported previously[1]. Modifications of our approach include laser driver and network analyzer optimizations to favor acquisition speed. To achieve this, the laser diodes remain on throughout the measurement to avoid start-up delays, and the network analyzer has a reduced number of measurement points for a given frequency sweep. In addition, we use the HTC Vive system to track the position of the probe in 3D space. To start an FDPM measurement, the laser driver enables all selected laser diodes (LD), while the network analyzer sequentially modulates each wavelength from the 1st to the nth laser diode. Laser light is guided to the sample by an optical fiber bundle and collected by an avalanche photodiode (APD) by direct contact or through an optical fiber. Amplitude and phase are then calculated by the network analyzer. For continuous-wave components, the spectrometer triggers the shutter on the broadband lamp. Broadband light is guided to the sample by an optical fiber bundle and collected by another optical fiber leading back to the spectrometer. Lastly, the HTC Vive Tracker attached to the probe wirelessly transmits data related to the probe position using infrared (IR) light emitting from the HTC Vive Base Station.

Reference

1. Pham TH, Coquoz O, Fishkin JB, Anderson E, Tromberg BJ. Broad bandwidth frequency domain instrument for quantitative tissue optical spectroscopy. *Rev Sci Instrum*. 2000;71(6):2500. doi:10.1063/1.1150665
